# Using prognostic and predictive clinical features to make personalised survival prediction in advanced hepatocellular carcinoma patients undergoing sorafenib treatment

**DOI:** 10.1038/s41416-019-0488-4

**Published:** 2019-06-11

**Authors:** Sarah Berhane, Richard Fox, Marta García-Fiñana, Alessandro Cucchetti, Philip Johnson

**Affiliations:** 1 Department of Biostatistics, University of Liverpool, Liverpool, UK; 2 Cancer Research UK Clinical Trials Unit, School of Cancer Sciences, University of Birmingham, Birmingham, UK; 3 Department of Medical and Surgical Sciences, Alma Mater Studiorum, University of Bologna, Bologna, Italy; 4 Department of Molecular and Clinical Cancer Medicine, University of Liverpool, Liverpool, UK

**Keywords:** Hepatocellular carcinoma, Targeted therapies, Liver cancer

## Abstract

**Background:**

Sorafenib is the current standard of care for patients with advanced hepatocellular carcinoma (aHCC) and has been shown to improve survival by about 3 months compared to placebo. However, survival varies widely from under three months to over two years. The aim of this study was to build a statistical model that allows personalised survival prediction following sorafenib treatment.

**Methods:**

We had access to 1130 patients undergoing sorafenib treatment for aHCC as part of the control arm for two phase III randomised clinical trials (RCTs). A multivariable model was built that predicts survival based on baseline clinical features. The statistical approach permits both group-level risk stratification and individual-level survival prediction at any given time point. The model was calibrated, and its discrimination assessed through Harrell’s c-index and Royston-Sauerbrei’s R^2^_D_.

**Results:**

The variables influencing overall survival were vascular invasion, age, ECOG score, AFP, albumin, creatinine, AST, extra-hepatic spread and aetiology. The model-predicted survival very similar to that observed. The Harrell’s c-indices for training and validation sets were 0.72 and 0.70, respectively indicating good prediction.

**Conclusions:**

Our model (‘PROSASH’) predicts patient survival using baseline clinical features. However, it will require further validation in a routine clinical practice setting.

## Background

The multikinase inhibitor, sorafenib, was the first agent shown to offer survival benefit to patients with advanced hepatocellular carcinoma (aHCC) in a prospective placebo-controlled clinical trial (The SHARP trial).^1^ Similar results were subsequently reported from the analogous Asia-Pacific study.^2^ Sorafenib remains^3^ the current standard of care for patients with aHCC in most countries offering a survival advantage of about three months compared to placebo. Although there are several potential new treatments for aHCC,^4^ sorafenib remains a first line treatment.^5^

Median overall survival (OS) for patients undergoing sorafenib treatment is about 10 months.^1,6,7^ However, at the individual patient level, there is a wide variation ranging from 3 months to more than 2 years.^8,9^As part of a personalised approach to sorafenib treatment, information on a patient’s baseline clinical features can potentially be utilised to make individual survival predictions. Previous studies looked at utility of current HCC staging systems in survival prediction but, none of them were found to be optimal.^10^ Even within the Barcelona Clinic Liver Cancer staging system, where sorafenib is the recommended treatment (BCLC C), better survival varied considerably according to baseline clinical features.^9^

Subsequent studies have examined prognostic factors affecting OS among sorafenib patients as well as other variables predictive of sorafenib OS benefit. Factors found to influence prognosis included neutrophil-to-lymphocyte ratio (NLR), alpha-fetoprotein (AFP), tumour size/stage, extra-hepatic spread (EHD), Child-Pugh score, aspartate aminotransferase (AST), compensated cirrhosis, ascites, macroscopic vascular invasion (MVI), performance status (PS), albumin and bilirubin levels.^9,11–16^ Variables that predict greater sorafenib benefit compared to placebo included lower neutrophil-to-lymphocyte ratio, absence of extra-hepatic spread (EHS) and being hepatitis C positive (HCV).^8,16–20^

In view of the aforementioned wide survival variation, we develop here for the first time a statistical model that predicts individual patient survival, using baseline clinical and laboratory features including those of prognostic and/or predictive significance.

## Methods

### Sorafenib clinical trials

We had access to the sorafenib control arms of two multinational phase III randomised clinical trials (RCT) that compared sorafenib with each of brivanib^6^ and sunitinib.^7^ The brivanib trial sorafenib arm consists of 588 patients with aHCC accrued between 2009 and 2011. The sunitinib trial sorafenib arm comprised 542 patients with aHCC, recruited between July 2008 and May 2010. Inclusion criteria (see Supplementary table 1) in both trials were very similar. All data were obtained with permission from Bristol-Myers Squibb and Pfizer respectively.

### Variables

Baseline variables available for analysis that were common to both trials are shown in Table 1. All variables were measured at baseline before the start of the treatment. Those used for model building were: age, sex, race, Eastern Cooperative Oncology Group (ECOG) score, creatinine, bilirubin, AST, AFP, albumin, international normalised ratio (INR), aetiology, tumour type (solitary/multiple), tumour size, presence of extra-hepatic spread and vascular invasion. Aetiology was categorised as HCV-related, hepatitis B virus (HBV)-related and “other”. The “other” group of aetiology also included patients with no known risk factors. Extra-hepatic spread included patients with lymph node involvement. Tumour number was presented as a binary variable (“solitary” or “multiple”) rather than discrete because there was more missing data in the latter (6.1% versus 11.2%). This discrepancy in missingness is due to some patients being marked as having multiple tumours rather than the actual number being given. Child-Pugh grade was excluded from the model building and replaced instead by its derivation components albumin, bilirubin and INR. The amount of missingness for each variable is summarised in Supplementary table 2. Patients with missing data in any of the listed variables above were excluded from the modelling analysis.

**Table 1:** Baseline characteristics

Variables	Sorafenib arm (*n* = 588)	Sorafenib arm (*n* = 542)
	Brivanib trial	Sunitinib trial
Age (years)	60 (12.19), *n* = 588	58 (12.94), *n* = 542
Sex (% male)	492 (83.67), *n* = 588	457 (84.32), *n* = 542
Race (%)	*n* = 588	*n* = 542
Asian	396 (67.35)	417 (76.94)
Caucasian	176 (29.93)	111 (20.48)
Black	9 (1.53)	10 (1.85)
Other	7 (1.19)	4 (0.74)
ECOG	*n* = 588	*n* = 539
0	352 (59.86)	289 (53.62)
1	236 (40.14)	250 (46.38)
Child-Pugh grade	*n* = 575	*n* = 542
A	529 (92.00)	542 (100.00)
B	46 (8.00)	0 (0.00)
C	0 (0.00)	0 (0.00)
Aetiology	*n* = 588	*n* = 532
HCV	112 (19.05)	106 (19.92)
HBV	254 (43.20)	276 (51.88)
Other	222 (37.76)	150 (28.20)
Tumour type (% multifocal)	424 (76.81), *n* = 552	353 (71.31), *n* = 495
Tumour number	3 (2, 4), *n* = 522	2 (1, 4), *n* = 495
Tumour size group (%)	*n* = 552	*n* = 542
< = 2 cm	72 (13.04)	84 (15.50)
>2 and < = 3 cm	69 (12.50)	78 (14.39)
>3 and < = 5 cm	111 (20.11)	92 (16.97)
>5 and < = 7 cm	78 (14.13)	87 (16.05)
>7 and < = 10 cm	78 (14.13)	91 (16.79)
>10 cm	144 (26.09)	110 (20.30)
Extra-hepatic spread (%)	421 (71.60), *n* = 588	352 (64.94), *n* = 542
Vascular invasion (%)	170 (28.91), *n* = 588	161 (30.55), *n* = 527
Creatinine (µmol/L)	74.26 (64.09, 86.63), *n* = 588	75.07 (63.00, 88.40), *n* = 492
Bilirubin (µmol/L)	13.68 (10.26, 20.52), *n* = 586	15.39 (10.43, 20.52), *n* = 489
AST (U/L)	57 (35, 93), *n* = 581	56 (35, 87), *n* = 489
AFP (ng/ml)	180.75 (8.50, 2984.30), *n* = 572	305.05 (13.00, 4000.00), *n* = 486
Albumin (g/l)	39 (5.21), *n* = 580	39 (5.04), *n* = 492
INR	1.09 (0.14), *n* = 557	1.08 (0.10), *n* = 469
Death (%)	419 (71.26), *n* = 588	386 (77.98), *n* = 495
Overall survival range, months (within those who died)	0.1 – 31.28, *n* = 419	0.4 – 31.35, *n* = 386
Median overall survival, months (95% CI)	9.77 (8.49, 11.51), *n* = 588	8.91 (7.89, 10.20), *n* = 495

*AFP* alpha-fetoprotein, *AST* aspartate aminotransferase, *ECOG* Eastern Cooperative Oncology Group, *g/l* grams per litre, *HBV* hepatitis B, *HCV* hepatitis C, *INR* international normalised ratio, *µmol/L* micromoles per litre

### Statistical methods

Analysis was carried out using Stata/SE 14.1 (StataCorp, Texas, USA). Continuous variables were reported as mean (with standard deviation [SD]) or median (with interquartile range [IQR]), the latter for variables with highly skewed distributions. Categorical variables were presented as counts and percentages. Continuous variables which exhibited extreme skewness were log transformed. Overall survival (OS) was measured from date of randomisation until date of death (any cause). Patients who were still alive were censored at their date of last follow-up. Overall survival curves for each dataset were plotted using the Kaplan–Meier (KM) method and median survival with 95% confidence interval (95% CI) were reported. Survival distributions were compared using hazard ratio (HR) and corresponding *p* values.

Univariable and multivariable analyses were undertaken using a flexible parametric survival model (see below). A multivariable model that predicts survival based on the baseline clinical features of patients was built using the sorafenib arm of the brivanib trial (training set). The sorafenib arm from the sunitinib trial was used as a validation set. The model was validated and tested according to the methodology suggested by Royston and Altman.^21^

#### Flexible parametric survival model

Flexible parametric survival regression was proposed by Royston and Parmar^22^ in 2002. It is an extension of the Weibull model, and models the log baseline cumulative hazard using restricted cubic splines. The mathematical basis of the model and its advantages over the traditional Cox regression is described in the supplementary appendix titled “*The flexible parametric survival model*” and Supplementary figure 1.

#### Model building and validation

Univariable analysis was undertaken to examine the prognostic influence of each individual variable. The hazard ratio, 95% CI and *p* values were reported. A multivariable model was then built using a stepwise backward selection of variables significant at the 5% level. Any strong interactions between the variables in the model were also examined. The hazard ratio, 95% CI and *p* values for the multivariable model were reported.

A time-dependent (TD) effect for each variable in the final model was sequentially added and tested using the likelihood ratio (LR) test to inspect for any proportion hazards violation. The optimal degrees of freedom (d.f.) or knots for the restricted cubic spline function was chosen by testing and comparing different d.f. using the LR test. The functional forms of the continuous variables were examined by plotting a smoothed curve through Martingale residuals estimates, with zero gradient signalling an appropriate form.

The linear predictor of the final model was then derived using its coefficients. In order to generate four risk categories, previously suggested cut-offs^21^ at the 15th, 50th and 85th centiles were applied to the linear predictor of the training set. Subsequent model predictions were grouped according to this classification. Individual-level survival prediction was undertaken by calculating the survival function at time t (i.e. probability of a patient surviving past time t). The method for deriving the survival function formula is described in the supplementary appendix.

The derived model was validated on the sorafenib arm of the second RCT (sunitinib trial). KM survival curves according to the risk categories were plotted and visually inspected for both the training and validation sets. Median OS and HR were also calculated for each risk category.

#### Model calibration

Model predictions according to the risk categories were visually inspected by overlaying the observed KM and predicted mean survival curves into one graph and examining how closely they agree. In addition to this, the corresponding observed versus predicted median survival as well as observed versus predicted percentage survival at 12 months were derived and reported. This was carried out for both training and validation sets.

#### Model discrimination

Model discriminative performance was measured using Harrell’s c-index^23^ and Royston-Sauerbrei’s *R*^2^_D_.^24^ Harrell’s c-index measures the proportion of patient pairs where the survival predictions and observed outcomes are in agreement with respect to rank. *R*^2^_D_ assesses the level of explained variation on the log relative hazard scale. Model parameters derived from the training set were first applied to the validation set before calculation of Harrell’s c-index and *R*^2^_D_. A higher value of Harrell’s c-index and *R*^2^_D_ is indicative of better model discrimination.

#### Missing data

In order to investigate the nature of missingness and its effect on the final model parameters, multiple imputation of missing data using chained equations^25–27^ was undertaken and coefficients and *p* values between the complete case final model and the one with the imputed data were compared for any divergence.

## Results

Both sorafenib arms had similar baseline features (Table 1) with the exception of the presence of Child-Pugh B patients (*n* = 46) in the brivanib trial patients (none in the sunitinib trial). Comparing the KM plots between the two datasets showed that there was no evidence of a statistical difference in survival (HR = 1.11, 95% CI: 0.97, 1.28, *p* = 0.128) (Supplementary figure 2).

### Univariable and multivariable analyses

Supplementary table 3 reports the hazard ratios, 95% CI and *p* values of the univariable flexible parametric survival models. They show that age, ECOG, aetiology, tumour size, extra-hepatic spread, vascular invasion, log(bilirubin), log(AST), log(AFP), albumin and INR were statistically significant prognostic factors.

Table 2 shows the variables that were selected for the final multivariable model, along with the hazard ratios, 95% CI and *p* values. The variables in the final model were vascular invasion, age, ECOG, log(AFP), albumin, log(creatinine), log(AST), extra-hepatic spread and aetiology, along with an interaction between age and vascular invasion.

**Table 2:** Multivariable flexible parametric regression - hazard ratio (with 95% CI) (*n* = 500)

Variables	Hazard ratio	*p* value
Vascular invasion		
No	1 (reference group)	
Yes	1.387 (1.087, 1.770)	0.008
Age centred at 60 years – No vascular invasion	0.977 (0.966, 0.989)	<0.0001
Age centred at 60 years – With vascular invasion	1.007 (0.992, 1.022)	0.353
ECOG		
0	1 (reference group)	
1	1.576 (1.263, 1.967)	<0.0001
Log (AFP (ng/ml))	1.087 (1.052, 1.123)	<0.0001
Albumin (g/l)	0.946 (0.925, 0.968)	<0.0001
Log (creatinine (µmol/L))	2.032 (1.301, 3.174)	0.002
Log (AST (U/L))	1.418 (1.172, 1.716)	<0.0001
Extra-hepatic spread		
No	1 (reference group)	
Yes	1.348 (1.038, 1.749)	0.025
Aetiology		
HCV	1 (reference group)	
HBV	1.692 (1.210, 2.366)	0.002
Other	1.661 (1.195, 2.308)	0.003
γ_1_	11.766 (7.554, 18.327)	<0.0001
γ_2_	1.120 (1.075, 1.167)	<0.0001
γ_0_ (constant)	2.84 × 10^−4^ (2.8 × 10^-5^, 2.87 × 10^−3^)	<0.0001

*γ*_*1*_ and *γ*_*2*_ are the basis functions of the restricted cubic spline (based here on 2 df). γ_0_ is log *λ*, where *λ* is the scale parameter.

*AFP* alpha-fetoprotein, *AST* aspartate aminotransferase, *ECOG* Eastern Cooperative Oncology Group, *g/l* grams per litre, *HBV* hepatitis B, *HCV* hepatitis C, *INR* international normalised ratio, *µmol/L* micromoles per litre.

**Fig. 1:**
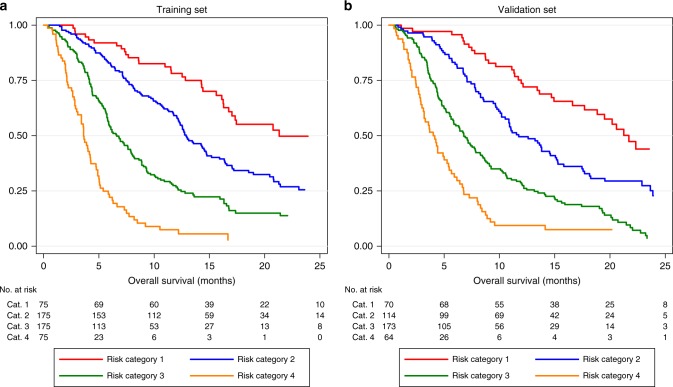
**a**, **b** Survival according to the risk categories as defined by the sorafenib model. Kaplan–Meier survival curves and the corresponding risk table for (**a**) training and (**b**) validation set

Partitioning the linear predictor using the prescribed cut-offs produced four distinct risk categories (ranked 1 to 4) in both the training and validation sets (Fig. 1a, b). The observed group-level median OS was comparable in both the training and validation sets, ranging approximately from 4 months in risk category 4 to 30 months in risk group 1.

The curves in Fig. 1 were generated as follows. First the linear predictor was derived:

$$\begin{array}{l}{\mathrm{Linear}}\,{\mathrm{predictor,}}\,{\mathrm{\eta }} = \left( {{\mathrm{0}}{\mathrm{.327}}\,{\times}\,{\mathrm{vascular}}\,{\mathrm{invasion}} \ast } \right) + \\ \left( { - {\mathrm{0}}{\mathrm{.0231}}\, \times \,\left( {{\mathrm{Age}} - {\mathrm{60}}} \right)} \right) + \\ \left( {{\mathrm{0}}{\mathrm{.0303}}\,{\mathrm{ \times }}\,\left[ {\left( {{\mathrm{Age}} - {\mathrm{60}}} \right){\times}\,{\mathrm{vascular}}\,{\mathrm{invasion}} \ast } \right]} \right){\mathrm{ + }}\\ \left( {{\mathrm{0}}{\mathrm{.455}}\,{\times}\,{\mathrm{ECOG}} \ast \!\ast } \right) + \\ \left( {{\mathrm{0}}{\mathrm{.0831}}\,{\times}\,{\mathrm{ln}}\left( {{\mathrm{AFP}}} \right)} \right) + \\ \left( {{\mathrm{ - 0}}{\mathrm{.0553}}\,{\times}\,{\mathrm{albumin}}} \right) + \\ \left( {{\mathrm{0}}{\mathrm{.709}}\,{\mathrm{x}}\,{\mathrm{ln}}\left( {{\mathrm{creatinine}}} \right)} \right) + \\ \left( {{\mathrm{0}}{\mathrm{.349}}\,{\times}\,{\mathrm{ln}}\left( {{\mathrm{AST}}} \right)} \right) + \\ \left( {{\mathrm{0}}{\mathrm{.298}}\,{\times}\,{\mathrm{extra}} {\hbox{-}} {\mathrm{hepatic}}\,{\mathrm{spread}} \ast } \right) + \\ \left( {\it{0.526}} \, \times \, {{HBV}} \ast \right) + \\ \left( {{\it{0}}{\it{.507}}\,{\times} \,{{other}}\,{{aetiology}}\,{{if}}\,{{not}}\,{{HCV}}/{{HBV}} \ast } \right)\end{array}$$

Where

$$\begin{array}{l}\ast {\mathrm{0}} = {\mathrm{no}}\,{\mathrm{and}}\,{\mathrm{1}} = {\mathrm{yes,}}\, \\ \ast \!\ast {\mathrm{0}} = {\mathrm{ECOG}}\ 0 \ {\mathrm{and}}\,{\mathrm{1}} = {\mathrm{ECOG}} \, 1\end{array}$$

HCV is the reference group

The following cutoffs were then applied to the linear predictor, η, to generate the four risk categories: ≤2.898 (risk category 1), >2.898 to ≤3.666 (risk category 2), >3.666 to ≤4.559 (risk category 3) and >4.559 (risk category 4).

To calculate the survival function for an individual patient at time t, the following three steps were undertaken. The derivation of these equations is explained in more detail in the supplementary.

(1) The log cumulative baseline hazard (spline function) at time *t* was derived as follows:

$${{s}}\left( {{\mathrm{log}}\,{{t}}} \right) = - {\mathrm{8}}{\mathrm{.167}} + {\mathrm{2}}{\mathrm{.465}}\,{\mathrm{log}}\,{{t}} + {\mathrm{0}}{\mathrm{.113}}\,{{z}}_{\mathrm{2}}$$

where ${{{z}}_{\mathrm{2}} = \left( {{\mathrm{log}}\,{{t}} - {\mathrm{1}}{\mathrm{.833}}} \right)_ + ^3 - {\mathrm{0}}{\mathrm{.361}}\,\left( {{\mathrm{log}}\,{{t}} + {\mathrm{1}}{\mathrm{.017}}} \right)_ + ^3 - {\mathrm{0}}{\mathrm{.639}}\,\left( {{\mathrm{log}}\,{{t}} - {\mathrm{3}}{\mathrm{.443}}} \right)_ + ^3}$ The “+” notation denotes (*x*)_+_  = max(0, *x*)

(2) Baseline survival function, at time t, was expressed as:

$${{S}}_{\mathrm{0}}\left( {{t}} \right) = {\mathrm{exp}}\left( { - {\mathrm{exp}}\left( {{{s}}\left( {{\mathrm{log}}\,{{t}}} \right)} \right)} \right)$$

(3) Survival function, S(t), at time t for an individual subject can then be calculated by:

$${{S}}\left( {{t}} \right) = {{S}}_{\mathrm{0}}\left( {\mathrm{t}} \right)^{{\mathrm{exp(\eta )}}}$$

where *η* is the linear predictor.

The values for S_0_(t) at time points 3, 6, 12, 18 and 24 months were 0.997, 0.991, 0.977, 0.965 and 0.955 respectively. For other time points, S_0_(t) can be calculated by following Steps 1 and 2.

For example, a patient with the following baseline features: No vascular invasion, 71 years of age, ECOG 1, AFP 850.3 ng/ml, albumin 46 g/l, creatinine 35.36 μmol/l, AST 29 U/l, no EHD and “other” aetiology (non-HBV, non-HCV) will have a survival function of 97%, 90%, 77%, 67% and 59% at times 3, 6, 12, 18 and 24 months, respectively.

An online calculator to generate the survival predictions at a group and individual level is available at: https://jscalc.io/calc/oGSDLHDsDg9g2XBF

### Model calibration

**Fig. 2:**
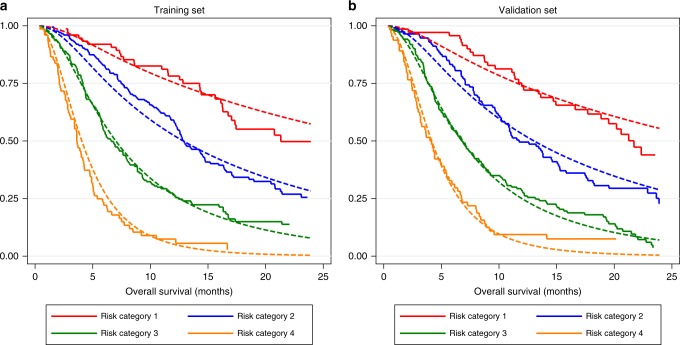
**a**, **b** Calibration plots. Comparing observed KM curves (solid line) and model-predicted mean survival curve (dashed line) for each risk category in the (**a**) training and (**b**) validation set

Observed KM and model-predicted survival curves according to the risk categories were closely matched in both the training and validation sets (Fig. 2a, b). This was also reflected by the similarities between the observed and predicted median OS as well as observed and predicted percentage survival at 12 months (Table 3) in both datasets. There was some discrepancy, however, between the observed and predicted median OS in the lowest risk category of the training set although the percentage survival at 12 months was almost identical (78 vs. 76%). Table 3 shows the median OS, percentage survival at 12 months, hazard ratio and *p* value according to each risk category.

**Table 3:** Predicted versus observed survival

Dataset	Risk category	N	Observed median OS (95% CI)	Predicted median OS (95% CI)	Observed % survival at 12 months (95% CI)	Predicted % survival at 12 months (95% CI)	Hazard ratio (95% CI)	*p* value	Harrell’s C index (95% CI*)	Royston-Sauerbrei’s R^2^_D_ (95% CI*)
Training	1	75	21.32 (16.28, NA)	30.99 (24.80, NA)	78.19 (66.85, 86.04)	75.99 (71.17, 81.14)	1	Reference	0.72 (0.69, 0.75)	0.27 (0.22, 0.36)
	2	175	12.89 (12.17, 14.70)	12.89 (11.68, 14.70)	59.09 (51.37, 65.99)	52.71 (48.32, 57.49)	1.99 (1.34, 2.93)	0.001		
	3	175	6.61 (5.79, 7.89)	7.17 (6.55, 7.86)	26.78 (20.30, 33.66)	26.66 (23.08, 30.79)	3.76 (2.55, 5.55)	<0.0001		
	4	75	3.62 (2.99, 4.24)	4.14 (3.65, 4.74)	7.44 (2.79, 15.18)	5.87 (3.62, 9.51)	9.69 (6.30, 14.91)	<0.0001		
Validation	1	70	21.71 (17.70, 25.66)	29.28 (23.55, NA)	73.64 (61.43, 82.51)	74.23 (69.38, 79.42)	1	Reference	0.7 (0.67, 0.73)	0.18 (0.11, 0.27)
	2	114	11.74 (10.20, 14.61)	13.13 (11.64, 15.07)	49.55 (40.04, 58.35)	53.85 (49.41, 58.68)	1.93 (1.29, 2.87)	0.001		
	3	173	6.78 (5.63, 7.86)	6.78 (6.32, 7.73)	28.70 (21.99, 35.74)	25.24 (21.29, 29.93)	3.73 (2.56, 5.44)	<0.0001		
	4	64	4.01 (2.99, 5.26)	4.24 (4.01, 4.90)	9.38 (3.82, 17.98)	5.83 (3.45, 9.85)	7.08 (4.61, 10.87)	<0.0001		
All	1	145	21.32 (17.70, 31.35)	30.39 (23.85, NA)	75.94 (67.98, 82.19)	74.84 (70.00, 80.02)	1	Reference	0.71 (0.69, 0.73)	0.22 (0.17, 0.29)
	2	289	12.76 (11.74, 14.31)	13.26 (11.51, 14.87)	55.29 (49.33, 60.84)	52.83 (48.45, 57.60)	1.93 (1.46, 2.54)	<0.0001		
	3	348	6.78 (5.89, 7.63)	6.91 (6.32, 7.63)	27.74 (23.02, 32.64)	25.82 (22.14, 30.11)	3.68 (2.81, 4.81)	<0.0001		
	4	139	3.68 (3.19, 4.28)	4.14 (3.68, 4.74)	8.32 (4.42, 13.77)	5.54 (3.35, 9.14)	8.16 (6.03, 11.04)	<0.0001		

NA = Upper limit of the 95% confidence interval did not intersect with 50% survival so cannot be estimated. Confidence intervals estimated from 200 bootstrap samples*

### Model discrimination

There was a slight fall in the Harrell’s c-index (from 0.72 to 0.70) and *R*^2^_D_ (from 0.27 to 0.18) in the validation set compared to the training set (Table 3); signalling a small deterioration in predictive power of the model. However, both figures were indicative of good prediction.

### All patients combined

**Fig. 3:**
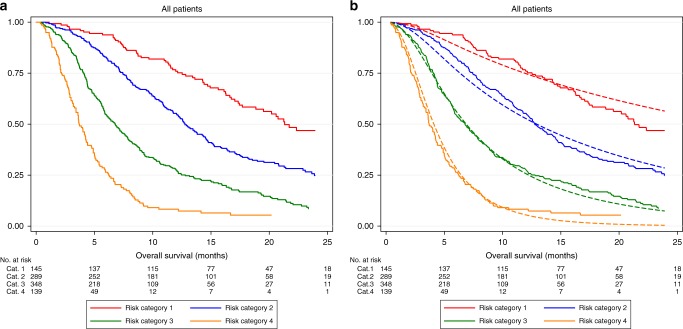
**a**, **b**. Survival and calibration plots for all patients combined. (**a**) Kaplan–Meier survival curves according to the risk categories as defined by the sorafenib model and (**b**) calibration plots of observed KM curves (solid line) and model-predicted mean survival curve (dashed line). Risk tables for both graphs shown below each figure

Since both the training and validation sets showed similar survival both overall and within each risk category, they were merged and KM survival curves (Fig. 3a) and calibration plots (Fig. 3b) were generated.

Observed percentage survival at 12 months for patients within risk categories 1 to 4 were 76%, 55%, 28% and 6% respectively, with corresponding HR (in comparison to risk category 1) being 1.93 (95% CI: 1.46, 2.54; *p* < 0.0001), 3.68 (95% CI: 2.81, 4.81; *p* < 0.0001) and 8.16 (95% CI: 6.03, 11.04; *p* < 0.0001). Model-predicted and observed survival were very similar, with the exception of some discrepancy in median OS in the lowest risk category, as mirrored in the individual training and validation set results. Table 3 shows the median OS, hazard ratio and *p* value when the model is applied to all the patients combined.

Finally, comparing the parameters between the complete case model and the one using imputed data showed very similar coefficients and *p* values (Supplementary table 4), therefore indicating that the final model was not greatly affected by missing data.

## Discussion

Although the SHARP trial and the Asia-Pacific study^1,2^ clearly demonstrated a significant improvement in survival over placebo in patients with advanced HCC, the absolute improvement in median survival was less than 3 months. Furthermore, as we show here, there is heterogeneity around the median survival figures. In this study, where patient inclusion criteria were analogous to that of the SHARP trial, survival ranged from less than one month to more than two years. Others have found similar heterogeneity.^8,9^ At present the recommended indication for sorafenib treatment is patients with well-preserved liver function and being unsuitable for loco-regional therapies,^5^ and this does not take into account likely survival. These figures are put into further context by a recent review of sorafenib amongst over 1000 Medicare beneficiaries^28^ noting that ‘survival is exceptionally short…..and downsides of sorafenib use - high drug-related symptom burden and high drug cost - must be considered in light of this minimal benefit’. To overcome this issue, in this paper, we have developed a statistical model [henceforth known as PROSASH (**PR**ediction **O**f **S**urvival in **A**dvanced **S**orafenib-treated **H**CC] that allows personalised survival predictions with a view to aiding patient counselling and trial design.

Using data collected for regulatory purposes with similar criteria to that used for the SHARP trial and Asia-Pacific study,^1,2^ we show that it is possible to predict survival on the basis of clinical features available at the time of diagnosis. Within the entire cohort of patients, we identified four risk categories (Fig. 1) whose median overall survival ranged from 4 months in the highest risk category to over 20 months in the lowest risk category (Table 3). The corresponding percentage survival at 12 months for risk categories 1 to 4 were approximately 8%, 28%, 55%, and 76% respectively (Table 3). This stratification in addition to the individual patient predictions, permits ‘personalisation’ of sorafenib therapy and may be useful in clinical trials to optimally stratify patients where sorafenib is the appropriate control arm. Thus, it would be possible to ensure that patients in a randomised phase III trial would have equivalent and matching prognostic features. Furthermore, the median survival figures in the higher risk groups are, in fact, worse than in the control (placebo) group of the SHARP study and this may lead clinicians to consider if the toxicity consequent on sorafenib therapy is worth any small potential survival benefit.

The model also offers insight into some of the factors that influence survival (Table 2). Notably, using HCV as the reference aetiology, the prognosis is clearly much worse in the HBV and ‘other’ groups. This is consistent with recent findings both from a retrospective review of the SHARP trial and Asia-Pacific study^1,2,16^ and meta-analysis studies,^8,20^ which identify HCV positivity as a key *predictive* factor for benefit after sorafenib. Our model thus contains the major factors that have been found to be either predictive of sorafenib benefit such as extra-hepatic spread, or prognostic, such as vascular invasion and AFP in the combined analysis of the SHARP and Asia-Pacific trials^16^ apart from NLR, which was not a recorded in our datasets. Although such data permits optimisation of the patient groups in whom sorafenib is administered, molecular markers related to the mechanism of action of targeted agents remain an important and, as yet unfulfilled, goal.^29^

The inclusion of aetiological factors in our model is clearly justified on the basis of the previously mentioned evidence that HCV positivity is a predictor of survival benefit compared to placebo. However, the inclusion of aetiological factors other than HBV and HCV, which are relatively ‘objective’ is problematical. Thus, the lifetime consumption of alcohol is very difficult to record accurately and when both alcohol and a type of viral hepatitis are recorded, attribution to one specific aetiology becomes highly speculative. The diagnosis of NAFLD is equally difficult in the setting of HCC since evidence of NAFLD has often disappeared by the time the patient develops cirrhosis and HCC.^30,31^ In the light of these observations we believe that categorisation aetiology as HCV, HBV or ‘other’ is the fairest option.

Another limitation of our study is that we did not have sufficient data to take into account pre- and post- study treatments. Since these are not predicated in the trial protocol, there is a wide variation in the treatment options and their duration, which makes it difficult to model statistically.

Since the guidelines for sorafenib treatment are based on clinical trial data it seems reasonable to build our prognostic model on similar datasets. Nonetheless, it will be important to validate the performance of the model in larger datasets and in patients treated in the routine clinical practice setting. Although the predicted survival in our model was very close to that observed at each risk category (Fig. 2), there was some discrepancy between the observed and predicted median survival in the low risk category of the training set towards the end of the follow-up period (after about 15 months). Possible explanations for this discrepancy may be due to the small number of patients within that group surviving beyond 20 months such that (a) there was not enough information for the model to correctly extrapolate survival for and (b) such patients may have different features (compared to others) and factors that affect their survival may not be accounted for by the model. The observed and predicted percentage survival at 12 months in this category was, however, very close (78 vs 76%).

We believe that the statistical approach adopted here could be used to generate a ‘virtual control group’ for phase II, single arm, trials of new agents. Thus, we can generate survival curves that predict the outcome of patients in such trials *had they received sorafenib* although quantitative estimation of differences between the trial arms (sorafenib predicted vs actual new agent) remains methodologically challenging. Such an approach might be a useful preliminary screen for new agents when a go /no go decision concerning progression from phase II to phase III has to be made.

## Supplementary information

Supplementary file

## Data Availability

All data remain the property of the sponsors of the trials from which they were extracted, as noted in the acknowledgments section.
