# Supplementary material for: Using prognostic and predictive clinical features to make personalised survival prediction in advanced hepatocellular carcinoma patients undergoing sorafenib treatment
Source: Br J Cancer. 2019 Jun 11;121(2):117–24. doi: 10.1038/s41416-019-0488-4 (PMC6738086; doi:10.1038/s41416-019-0488-4)
Supplement: Supplementary file 1 — Supplementary file [file 41416_2019_488_MOESM1_ESM.docx]

**SUPPLEMENTARY MATERIAL**

**Supplementary appendix**

**The flexible parametric survival model**

*Concept and development*

The description of the flexible parametric survival model below is based on a book by Royston and Lambert^1^.

Survival analysis is often carried out using Cox regression modelling. It was first introduced in 1972^2^. Its main assumption is proportional hazards where the relative hazard between any two treatment groups is constant over time. It can be algebraically written as:

h_i_(t|x_i_) = h_0_(t) exp(x_i_β) (1)

h_i_(t|x_i_) is the hazard function of the *i*th individual conditional on covariates x_i_. β is the vector of the regression coefficients and h_0_(t) is the baseline hazard function when all the covariates x_i_ equals to zero. In a Cox model, the shape of the baseline hazard is left unspecified. Hence the Cox model is referred to as being a semi-parametric model.^3^ In many settings, the advantage of the Cox model over other parametric models like exponential and Weibull is its lack of specification of the baseline hazard function.

However, this lack of specification of the baseline hazard function in the Cox model comes with some setbacks^4^. Firstly, by ignoring the baseline hazard, we are missing out on important information that may contribute to a better prediction of survival. Secondly, there is no widely accepted method of dealing with violation of the proportional hazards assumption in Cox models and thirdly, lack of baseline hazard specification means that the model is too closely matched to the data that it is based on and its inherent features, increasing the risk of over-fitting. Over-fitting is a disadvantage when trying to create a risk-prediction model that generalises well to other scenarios. A parametric approach also allows (cautious) extrapolation of survival estimates outside the study observation time and this is not possible with a Cox model.

The above reasons make a case for using parametric survival models like the exponential and Weibull models. However, these are not flexible enough^5^ as they make strong assumptions about the shape of the baseline hazard function. For instance, the Weibull model assumes that the baseline hazard monotonically increases, decreases or remains constant (equivalent to the exponential model), whereas “real-life” data may show that hazards peak at certain points in time, followed by a decline.

Issues with the Cox and the standard parametric models highlighted above led Royston and Parmar^1,4^ to develop a new more flexible class of parametric survival models that extends the standard parametric models, resulting in a better fit to the real data. This was done with the aid of restricted cubic splines. Cubic splines are polynomial segments (β_0_ + β_1_x + β_2_x^2^ + β_3_x^3^) that smoothly join at intervals called “knots”. At the knot locations, cubic functions are forced to join resulting in a continuous function with flexibility incorporated. Two of the models developed by Royston and Parmar^4^ in 2002 were the flexible parametric proportional hazards and proportional odds models. In this study, we use the flexible parametric proportional-hazards model which is an extension of the Weibull model. It models the baseline cumulative hazard.

The baseline hazard function of the Weibull model is written as:

h_0_(t) = $\lambda\gamma_{1}t^{\gamma_{1}-1}$ (2)

where γ_1_ is the shape parameter, λ is the scale parameter and t is time.

The log cumulative baseline hazard is written as:

log H_0_(t) = log λ + γ_1_log t = γ_0_ + γ_1_log t (3)

where log H_0_(t) is linear function of log t

The above can be extended by including a restricted cubic spline function of log t:

log H_0_(t) = γ_0_ + γ_1_log t + γ_2_z_1_ (log t) + γ_3_z_2_ (log t) + ……. (4)

Log t, z_1_(log t) and z_2_(log t) are the basis functions of the restricted cubic spline. The number of knots or degrees of freedom specifies the number of basis functions. The derivation of these basis functions is described below.

We first define m interior knots (k_1_, …, k_m_) in addition to the two boundary knots, k_min_ and k_max_ which are placed at the minimum and maximum of log t respectively. m interior knots has m+1 degrees of freedom and m+1 basis functions.

Equation 4 can also be rewritten as:

s(log t *| γ*) = γ_0_ + γ_1_z_1_ + γ_2_z_2_ + γ_3_z_3_ + γ_m+1_z_m+1_ (5)

z_j_ variables are derived as follows:

z_1_ = log t (6)

z_j_ = ${(log t - k_{j})}_{+}^{3}$ – $\lambda_{j}{(log t - k_{\min})}_{+}^{3}$ – $(1-\lambda_{j}){(log t - k_{\max})}_{+}^{3}$ (7)

where for j=2, …, m+1,

$\lambda_{j}$ = $\frac{k_{\max}- k_{j}}{k_{\max}- k_{\min}}$ (8)

Hence, a baseline spline function with three interior knots (in addition of two boundary knots) can be written as,

s(log t *| γ*) = γ_0_ + γ_1_z_1_ + γ_2_z_2_ + γ_3_z_3_ + γ_4_z_4_ (9)

Two interior knots: s(log t *| γ*) = γ_0_ + γ_1_z_1_ + γ_2_z_2_ + γ_3_z_3_ (10)

One interior knot: s(log t *| γ*) = γ_0_ + γ_1_z_1_ + γ_2_z_2_ (11)

No knots: s(log t *| γ*) = γ_0_ + γ_1_z_1_ (which goes back to the Weibull model) (12)

Survival function, S(t), at time t for an individual subject can then be defined as:

S(t) = S_0_(t)^exp(η)^ (13)

where S_0_(t) is the baseline survival function = exp(-exp(s(log t *| γ*))) and η is the linear predictor of the model.

Spline functions make the Weibull model more flexible to accommodate the survival distributions, rather than restriction to just monotonically increasing or decreasing trend.

*Reasons for choosing the flexible parametric survival model*

- **Easier survival predictions:** By harvesting information from the baseline hazard, the flexible parametric model can predict survival more easily.^6^
- **Extrapolation of survival estimates:** Unlike the Cox model, the flexible parametric model allows extrapolation of survival estimates outside the study observation time. This means that it can predict survival outside the time range of the model that it was based on. However, accuracy of this prediction depends on the assumed (modelled) survival distribution in the upper tail of follow-up time.
- **More flexible than other parametric models:** the flexible parametric models are more flexible than other parametric models such as Weibull and exponential as it does not impose strong assumptions on the baseline hazard.
- **Less sensitive to random variations due to sparse data:** Since the fit from the Cox model very closely matches the data, it picks up artefacts (due by sparse data) that are specific features to the data it is based on, whilst the flexible parametric regression models the overall trend of the baseline hazard function without picking up random variations. Supplementary figure 1 *(reproduced and modified from “Flexible Parametric Survival Analysis Using Stata Beyond The Cox Model”^1^: Chapter 1, page 4)* shows differences in the hazard function fitted by the flexible parametric model and the Cox regression model.^1^ The curves from the Cox model were not smooth thus making them hard to interpret compared to the flexible parametric model.^1^ The latter does not pick up the sharp increase in the mortality rate post 4.5 years caused by a small number of deaths at the end of follow-up having an undue influence on the mortality rate whereas the Cox model does. Whilst this will not cause problems when calculating hazard ratios (since these sparse points will not be given much emphasis), it is an issue when calculating absolute risk as such points will lead to “wild” estimates, particularly in situations where a small number of patients remain at risk at the end of the study period.

For the above reasons, as well as evidence from past studies that showed a preference for flexible parametric models over Cox models for the prediction of survival^7,8^ led to the decision to use flexible parametric survival regression rather than the Cox model to build our statistical model.

**Supplementary tables**

| **Supplementary table 1:** Inclusion criteria | |
| --- | --- |
| **Brivanib vs Sorafenib trial** (from Johnson et al) | **Sunitinib vs Sorafenib trial** (from Cheng et al) |
| - Men and women ages 18 years or older - Histologically or cytologically confirmed, advanced HCC – advanced disease was defined as disease not eligible for or progressive after surgical or locoregional therapy - No prior systemic therapy for HCC - Locoregional therapy must have been completed at least 3 weeks before the baseline scan - At least one untreated measurable lesion by MRI or spiral CT - Cirrhotic status of Child-Pugh class A - Eastern Cooperative Oncology Group (ECOG) performance status 0 or 1 - Life expectancy of at least 12 weeks - Adequate hematologic function with absolute neutrophil counts ≥1500/µL, platelet count ≥60x10^9^/L and haemoglobin ≥8.5 g/dL - Adequate hepatic function with serum total bilirubin ≤ 3 mg/dL, serum albumin ≥2.8 g/dL, and ALT and AST ≤5 x the institutional ULN - Adequate renal function with serum creatinine ≤2.0 mg/dL - Amylase and lipase <1.5 x ULN - INR ≤ 2.3 or PT ≤ 6 seconds above control | - Patients at least 18 years old - Histologically confirmed, locally advanced or metastatic HCC and were candidates for systemic anticancer treatment - Measurable disease according to Response Evaluation Criteria in Solid Tumours - Cirrhotic status Child-Pugh A - Eastern Cooperative Oncology Group (ECOG) performance status 0 or 1 - Adequate hepatic, renal, and hematologic function: neutrophils ≥1500/µL; platelets ≥75000/µL; serum AST and ALT ≤ 5 x the upper limit of normal; and total bilirubin <2mg/dL or ≤3mg/dL with albumin >3.5g/dL |

| **Supplementary table 2:** Number (and percentage) missing data | | |
| --- | --- | --- |
|  | **Sorafenib (n=588)** | **Sorafenib (n=542)** |
| **Variables** | **Brivanib trial** | **Sunitinib trial** |
| **Age** | 0 | 0 |
| **Sex** | 0 | 0 |
| **Race** | 0 | 0 |
| **ECOG** | 0 | 3 (0.55) |
| **Aetiology** | 0 | 10 (1.85) |
| **Tumour type (solitary/multiple)** | 36 (6.12) | 47 (8.67) |
| **Tumour size group** | 36 (6.12) | 0 |
| **Extrahepatic spread** | 0 | 0 |
| **Vascular invasion** | 0 | 15 (2.77) |
| **Creatinine (µmol/L)** | 0 | 50 (9.23) |
| **Bilirubin (µmol/L)** | 2 (0.34) | 53 (9.78) |
| **AST (U/L)** | 7 (1.19) | 53 (9.78) |
| **AFP (ng/ml)** | 16 (2.72) | 56 (10.33) |
| **Albumin (g/l)** | 8 (1.36) | 50 (9.23) |
| **INR** | 31 (5.27) | 73 (13.47) |
| **Missing any** | 88 (14.97) | 143 (26.38) |
| Variables in the final model shaded in grey | | |

| **Supplementary table 3:** Univariable flexible parametric regression based on the brivanib trial data - hazard ratio (with 95% CI) (n=500) | | | |
| --- | --- | --- | --- |
| **Variables** | **Hazard ratio (95% CI)** | **p-value (Wald test)** | **p-value (LR test)** |
| **Age (years)** | 0.985 (0.977, 0.994) | 0.001 | 0.001 |
|  |  |  |  |
| **Sex** |  |  |  |
| Female | 1 |  |  |
| Male | 1.074 (0.808, 1.429) | 0.622 | 0.619 |
|  |  |  |  |
| **Race** |  |  |  |
| Asian | 1 |  | 0.150 |
| Caucasian | 0.770 (0.611, 0.970) | 0.027 |  |
| Black | 1.067 (0.440, 2.588) | 0.885 |  |
| Other | 0.752 (0.241, 2.347) | 0.624 |  |
|  |  |  |  |
| **ECOG** |  |  |  |
| 0 | 1 |  |  |
| 1 | 1.747 (1.416, 2.156) | <0.0001 | <0.0001 |
|  |  |  |  |
| **Aetiology** |  |  |  |
| HCV | 1 |  | 0.001 |
| HBV | 1.712 (1.279, 2.292) | <0.0001 |  |
| Other | 1.409 (1.041, 1.907) | 0.026 |  |
|  |  |  |  |
| **Tumour type** |  |  |  |
| Solitary | 1 |  |  |
| Multifocal | 1.198 (0.937, 1.532) | 0.149 | 0.143 |
|  |  |  |  |
| **Tumour size group** |  |  |  |
| <=2cm | 1 |  | 0.009 |
| >2 and <=3cm | 0.952 (0.615, 1.473) | 0.825 |  |
| >3 and <=5cm | 1.286 (0.880, 1.881) | 0.194 |  |
| >5 and <=7cm | 1.517 (1.021, 2.254) | 0.039 |  |
| >7 and <=10cm | 1.684 (1.123, 2.526) | 0.012 |  |
| >10cm | 1.599 (1.118, 2.287) | 0.010 |  |
|  |  |  |  |
| **Extra-hepatic spread** |  |  |  |
| No | 1 |  |  |
| Yes | 1.855 (1.462, 2.354) | <0.0001 | <0.0001 |
|  |  |  |  |
| **Vascular invasion** |  |  |  |
| No | 1 |  |  |
| Yes | 1.947 (1.562, 2.427) | <0.0001 | <0.0001 |
|  |  |  |  |
| **Log (creatinine (µmol/L))** | 1.336 (0.879, 2.031) | 0.175 | 0.175 |
| **Log (Bilirubin (µmol/L))** | 1.398 (1.156, 1.691) | 0.001 | 0.001 |
| **Log (AST (U/L))** | 1.659 (1.403, 1.962) | <0.0001 | <0.0001 |
| **Log (AFP (ng/ml))** | 1.107 (1.072, 1.143) | <0.0001 | <0.0001 |
| **Albumin (g/l)** | 0.941 (0.921, 0.961) | <0.0001 | <0.0001 |
| **INR** | 2.214 (1.078, 4.549) | 0.030 | 0.041 |
| Abbreviations: AFP, alpha-fetoprotein; AST, aspartate aminotransferase; ECOG, Eastern Cooperative Oncology Group; g/l, grams per litre; HBV, hepatitis B; HCV, hepatitis C; INR, international normalised ratio; LR, likelihood ratio; µmol/L, micromoles per litre. | | | |

| **Supplementary table 4:** comparing coefficients between the complete case and imputed data models | | | | |
| --- | --- | --- | --- | --- |
| **Variables** | **Complete case (n=500)** | | **Imputation (n=588)** | |
|  | **β-estimates** | **p-value** | **β-estimates** | **p-value** |
| **Vascular invasion** |  |  |  |  |
| No | 1 (reference group) |  | 1 (reference group) |  |
| Yes | 0.327 (0.084, 0.571) | 0.008 | 0.370 (0.143, 0.597) | 0.001 |
|  |  |  |  |  |
| **Age centred at 60 years –** No vascular invasion | -0.023 (-0.035, -0.011) | <0.0001 | -0.019 (-0.030, -0.008) | <0.0001 |
| **Age centred at 60 years –** With vascular invasion | 0.007 (-0.008, 0.022) | 0.353 | 0.005 (-0.009, 0.019) | 0.494 |
|  |  |  |  |  |
| **ECOG** |  |  |  |  |
| 0 | 1 (reference group) |  | 1 (reference group) |  |
| 1 | 0.455 (0.233, 0.676) | <0.0001 | 0.484 (0.280, 0.688) | <0.0001 |
|  |  |  |  |  |
| **Log (AFP (ng/ml))** | 0.083 (0.050, 0.116) | <0.0001 | 0.077 (0.046, 0.109) | <0.0001 |
| **Albumin (g/l)** | -0.055 (-0.078, -0.033) | <0.0001 | -0.059 (-0.080, -0.038) | <0.0001 |
| **Log (creatinine (µmol/L))** | 0.709 (0.263, 1.155) | 0.002 | 0.811 (0.409, 1.213) | <0.0001 |
| **Log (AST (U/L))** | 0.349 (0.158, 0.540) | <0.0001 | 0.353 (0.176, 0.531) | <0.0001 |
|  |  |  |  |  |
| **Extra-hepatic spread** |  |  |  |  |
| No | 1 (reference group) |  | 1 (reference group) |  |
| Yes | 0.298 (0.037, 0.559) | 0.025 | 0.303 (0.057, 0.549) | 0.016 |
|  |  |  |  |  |
| **Aetiology** |  |  |  |  |
| HCV | 1 (reference group) |  | 1 (reference group) |  |
| HBV | 0.526 (0.191, 0.861) | 0.002 | 0.475 (0.158, 0.793) | 0.003 |
| Other | 0.507 (0.178, 0.836) | 0.003 | 0.393 (0.081, 0.705) | 0.014 |
|  |  |  |  |  |
| **γ_1_** | 2.465 (2.022, 2.908) | <0.0001 | 2.741 (2.203, 3.279) | <0.0001 |
| **γ_2_** | 0.113 (0.072, 0.154) | <0.0001 | 0.090 (0.057, 0.122) | <0.0001 |
|  |  |  |  |  |
| **γ_0_ (constant)** | -8.167 (-10.482, -5.853) | <0.0001 | -8.134 (-10.222, -6.046) | <0.0001 |
| *γ_1_* and *γ_2_* are the basis functions of the restricted cubic spline (based here on 2 df). γ_0_ is log λ where λ is the scale parameter.  Abbreviations: AFP, alpha-fetoprotein; AST, aspartate aminotransferase; ECOG, Eastern Cooperative Oncology Group; g/l, grams per litre; HBV, hepatitis B; HCV, hepatitis C; INR, international normalised ratio; µmol/L, micromoles per litre. | | | | |

**Supplementary figure legends**

**Supplementary figure 1 (see appendix)**: Hazard functions estimated under proportional hazards assumption by the Cox model and the flexible parametric survival model. (Reproduced and modified from “Flexible Parametric Survival Analysis Using Stata Beyond The Cox Model”^1^: Chapter 1, page 4).

**Supplementary figure 2:** KM plot comparing survival between the sorafenib arms of the two trials. Table shows median survival in months (with 95% CI).

**Supplementary figures**

**Artefact due to sparse data picked up by Cox model**

Supplementary figure 1

| **Group** | **N** | **Median OS, months (95% CI)** | **Hazard ratio (95% C.I.)** | **p-value** |
| --- | --- | --- | --- | --- |
| **Sorafenib (brivanib trial)** | 588 | 9.77 (8.49, 11.51) | 1 | Reference |
| **Sorafenib (sunitinib trial)** | 495 | 8.91 (7.89, 10.20) | 1.11 (0.97, 1.28) | 0.128 |

Supplementary figure 2

**Supplementary references**

1. Royston P, Lambert PC: Flexible parametric survival analysis using Stata: beyond the Cox model. 2011

2. СОХ D: Regression Models and Life-Tables. Journal of, 1972

3. Collett D: Modelling survival data in medical research, CRC press, 2015

4. Royston P, Parmar MK: Flexible parametric proportional-hazards and proportional-odds models for censored survival data, with application to prognostic modelling and estimation of treatment effects. Stat Med 21:2175-97, 2002

5. Crowther MJ, Lambert PC: A general framework for parametric survival analysis. Stat Med 33:5280-97, 2014

6. Lambert PC, Royston P: Further development of flexible parametric models for survival analysis. Stata Journal 9:265, 2009

7. Li B, Cairns JA, Robb ML, et al: Predicting patient survival after deceased donor kidney transplantation using flexible parametric modelling. BMC Nephrol 17:51, 2016

8. Ramezani Tehrani F, Mansournia MA, Solaymani-Dodaran M, et al: Flexible parametric survival models built on age-specific antimullerian hormone percentiles are better predictors of menopause. Menopause 23:676-81, 2016
